# Impact of a pilot mHealth intervention on treatment outcomes of TB patients seeking care in the private sector using Propensity Scores Matching—Evidence collated from New Delhi, India

**DOI:** 10.1371/journal.pdig.0000421

**Published:** 2024-09-11

**Authors:** Ridhima Sodhi, Vindhya Vatsyayan, Vikas Panibatla, Khasim Sayyad, Jason Williams, Theresa Pattery, Arnab Pal

**Affiliations:** 1 William J Clinton Foundation, New Delhi, India; 2 TB Alert India, New Delhi, India; 3 Disease Management Programs, Global Public Health at Johnson & Johnson, Germany; Iran University of Medical Sciences, ISLAMIC REPUBLIC OF IRAN

## Abstract

Mobile health applications called Digital Adherence Technologies (DATs), are increasingly used for improving treatment adherence among Tuberculosis patients to attain cure, and/or other chronic diseases requiring long-term and complex medication regimens. These DATs are found to be useful in resource-limited settings because of their cost efficiency in reaching out to vulnerable groups (providing pill and clinic visit reminders, relevant health information, and motivational messages) or those staying in remote or rural areas. Despite their growing ubiquity, there is very limited evidence on how DATs improve healthcare outcomes. We analyzed the uptake of DATs in an urban setting (DS-DOST, powered by Connect for Life^TM^, Johnson & Johnson) among different patient groups accessing TB services in New Delhi, India, and subsequently assessed its impact in improving patient engagement and treatment outcomes. This study aims to understand the uptake patterns of a digital adherence technology and its impact in improving follow-ups and treatment outcomes among TB patients. Propensity choice modelling was used to create balanced treated and untreated patient datasets, before applying simple ordinary least square and logistic regression methods to estimate the causal impact of the intervention on the number of follow-ups made with the patient and treatment outcomes. After controlling for potential confounders, it was found that patients who installed and utilized DS-DOST application received an average of 6.4 (95% C.I. [5.32 to 7.557]) additional follow-ups, relative to those who did not utilize the application. This translates to a 58% increase. They also had a 245% higher likelihood of treatment success (Odds ratio: 3.458; 95% C.I. [1.709 to 6.996]).

## Author summary

The research tries to understand the impact of using cost-effective digital adherence tools in improving treatment outcomes among patients diagnosed with drug-sensitive Tuberculosis (TB). As the treatment duration for TB is fairly long (at least 6 months) and complicated (multiple drugs, typically given in two distinct phases), there are challenges associated with ensuring treatment adherence. The research finds that digital tools such as a mobile application can be a useful aid, albeit only when they are used in conjunction with the support of a healthcare worker. The digital tool analyzed as part of this study, sent medication reminders to patients, and enabled healthcare workers to track patients’ adherence. This ensured that healthcare workers follow-up with their patients to ensure adherence, resulting in increased odds of getting a favourable treatment outcome. Further, the study underscores that a digital tool used in isolation may not result in improved outcomes among patients, highlighting the role of healthcare workers and need for tailored interventions. In conclusion, digital adherence technologies can act as cost-effective measure in empowering healthcare workers to support their patients and subsequently improve treatment outcomes.

## Introduction/background

Digital adherence technologies (DATs) have evolved over time; however, there is limited evidence evaluating their impact on treatment or patient management outcomes [1–3]. A previous study outlines multiple approaches of evidence generation for evaluating the efficacy of a mHealth solution, while also highlighting the inherent challenges [4]. One critical challenge highlighted is the rapid pace of development of technologies, which can lead developers to modify the intervention, making it difficult to accurately assess its impact. Among the approaches suggested for evaluating interventions, randomized controlled trials (RCTs) are the most common but typically take too long to yield results (5–7 years) [4]. Another recommended approach called CEEBIT (Continuous Evaluation of Evolving Behavioural Interventions) involves assessing multiple technologies and continuously eliminating those that are less effective [4,5].

Notwithstanding the challenges, multiple studies have emphasized the need for evaluating DATs, and a simultaneous lack of the same [2,6–8]. Digital adherence technologies (DATs), such as mobile health applications, electronic medication monitors, and video directly observed therapy (VDOT), have been employed globally to address the challenges of long and complex TB treatment regimens [1,3,8–10]. These technologies offer benefits, including timely medication reminders, easy access to health information, and remote monitoring of patient adherence, which are particularly valuable in resource-limited settings [8,10]. A systematic evaluation of the role of mobile health interventions in enhancing interventions in PPM (public- private mix) for TB care illustrated the increasing universe of such solutions [11]. However, none of the studies evaluate the precise impact of such technologies on patient management (encompassing one or all aspects of pill reminders, patient follow-ups, monitoring adherence, empowering patients with messages on disease awareness, side effects and health information) or treatment outcomes. Other studies on the usage of such technologies in TB care, also lack quantitative evidence of such technologies on improving disease management or behaviour modification [1,2].

Our study is aimed at closing this gap by assessing the impact of a pilot intervention with Connect for Life (CfL), a mobile-based digital adherence technology, on patient management and treatment outcomes. Additionally, our study contributes to the growing body of research on Digital Adherence Technologies. By leveraging a quasi-experimental design and propensity score matching, we aim to provide robust evidence on the effectiveness of DATs in a real-world, urban healthcare setting. The study utilizes propensity choice modelling to balance the test and control groups, thus enabling precise estimates of the impact on treatment outcomes. The natural limitation of this method is that the test and control groups can only be balanced for the covariates on which the data is available. However, the magnitude and significance of results obtained across different model specifications render confidence to the inferences.

## Methods

### Ethical approval

CHAI’s Scientific and Ethical Review Committee (SERC) waived informed consent as anonymized programmatic data was utilized for the study. Additionally, all individuals who enrolled in the CfL pilot provided informed consent prior to enrolment, both through a written statement and via the Interactive Voice Response System (IVRS). In the case of patients who were below 18 years of age, parents/guardians were primary participants, and provided written consent. The written consents were obtained through treatment coordinators and were reviewed by the project lead.

### DS-TB care services under Project JEET

The Joint Effort for Elimination of Tuberculosis (Project JEET) began in 2018 and is a large-scale private health sector engagement initiative for TB [12]. The services offered through the program are intended to reduce challenges which limit the Indian healthcare system in arresting TB transmission, facilitating access to appropriate TB care, and supporting TB patients throughout their treatment. As part of JEET, treatment coordinators liaise with private providers to facilitate the notification of newly diagnosed TB patients in a digital government tracking system called Nikshay. This notification helps in tracking diagnosed patients and offering them a package of services provided by the National Tuberculosis Elimination program (NTEP). The patients also receive regular counselling support from a designated treatment coordinator through in-person and telephonic follow-ups, along with quality assured diagnostic services (molecular Testing) and access to free government sponsored FDC (Fixed Dose Combination) drugs. The services are provided in close coordination with the treating physician. The program helps in limiting the onward transmission of disease through the combination of support described. At the time of the initiation of the CfL pilot (November 2019), approximately 900 private providers in New Delhi were engaged with Project JEET as part of the Patient Provider Support Agency (PPSA) managed by the William J. Clinton Foundation (WJCF).

### Pilot set up

The pilot was initiated in November 2019 in three private care facilities in New Delhi, India, namely, Vinod Karhana Hospital, Sir Ganga Ram Hospital, and St. Stephen’s hospital. The patients under this intervention were notified through Project JEET. The intervention was done in collaboration between the William J Clinton Foundation (WJCF), TB Alert India, and Johnson & Johnson (J&J). J&J developed and customized Connect for Life (CfL), a mobile application built to help treatment providers and healthcare workers in patient management. Newly diagnosed TB patients (and/ or their caregivers) were informed about the CfL application and consent was sought prior to enrolling them in the program. New patients were enrolled in the program between November 26, 2019, and March 15, 2020. Patients who consented were provided with digital support offered by the CfL mobile application and a designated treatment coordinator, in addition to the standard services provided to patients managed under Project JEET. Two key elements of the pilot are described below:

1. **Connect for Life**: This is a mobile, feature or smartphone-based health application system that utilizes a combination of IVRS (Interactive Voice Response System) and SMS (short messaging service) to help patients remember to take their medications, provide reminders for visiting the clinic for planned check-ups and medication refills, and give them health tips covering topics such as nutrition, the significance of adherence, stigma, managing adverse drug reactions, and community transmission. As of 2021, CfL is an Open-Source Platform [13] and can be downloaded and used by any organization or country. Some key features of the mHealth application include:
  - Facility to enroll both the patient and the caregiver
  - Modify the frequency of the pill/health-tip reminders between daily, weekly, or monthly, and set up a preferred time to receive reminders
  - Dashboards to track adherence data by the healthcare worker, enabling better monitoring and follow up by healthcare worker
  - Option to opt out of service at any time during the treatment
2. **Treatment Coordinators**: Three healthcare workers were engaged for the pilot, each responsible for managing patients enrolled in one of the three private facilities under the project. While all patients under Project JEET are assigned a treatment coordinator, these three treatment coordinators were a specifically trained in using the mobile application and explaining its functions and utilities to patients and caregivers. Once patients consented to participate in the pilot, the treatment coordinator activated the patient’s mobile telephone number with a unique password and explained how to use the phone and/or text messaging functionalities. These treatment coordinators had access to the CfL visualization dashboard, which enabled them to monitor the self-recorded medication adherence. This helped treatment coordinators provide differentiated counselling to patients, by optimizing the follow ups based on dashboard analytics, which potentially reduced their workload.
3. **Training of the treatment coordinators**: The three treatment coordinators engaged for the pilot were trained by the WJCF staff. Except for the additional training on CfL, the rest of their training was similar to other treatment coordinators under Project JEET.

### Study design

The quasi-experimental study compared the follow-up regimen and treatment outcomes of patients who participated in the CfL pilot (test dataset) with those patients not part of the pilot, but notified during the same period in the same these three facilities (control dataset). Both the test and control datasets were matched using propensity scores to ensure robust measures.

### Data source(s)

There are two data sources utilized for this study. The first is data obtained from the CfL application, which consisted of information on 476 patients enrolled in the pilot. The second source comprises programmatic data collected as part of services rendered under Project JEET. This encompassed data collected as part of regular JEET operations and includes 1) Demographic characteristics of TB patients such as age, sex, and diagnosing district, 2) TB diagnostic and treatment information including type of diagnostic test, pulmonary or extrapulmonary diagnosis, provision of free drugs, treatment outcomes, and 3) number of follow-up contacts made by treatment coordinators. The data collected by the pilot intervention included information on the utilization of the CfL application. This consisted of multiple metrics such as the preferred time for health tips delivery for each patient, whether they recorded the adherence, and the frequency of interactions with the IVRS for various services. Data on consent to participate in the pilot and engagement level with CfL was also captured in the CfL application.

### Data selection (Inclusion and Exclusion criteria)

We considered data for patients enrolled in one of the three facilities where the pilot was conducted and were diagnosed with drug sensitive TB between 1^st^ October 2019 and 31^st^ March 2020. This also represents patients who were diagnosed before the COVID-19 pandemic started impacting health services operations in India (the first nation-wide lockdown in India was implemented on 24^th^ of March 2020). Among these patients, only those with an assigned treatment outcome at the time of the study were included. The selection criteria are described in more detail in Table 1 and 2. A total of 989 patients were enrolled, out of which 276 enrolled in the CfL application, provided consent, and utilized it throughout their treatment.

**Table 1:** CfL Test dataset: Selection criteria pathway.

Reason for inclusion/exclusion	#Patients Excluded	Patients retained
Patients enrolled	(+) 476	476
Consent required	(-) 138	338
Patients opted out of the application after using	(-) 18	320
Patient died before treatment initiation	(-) 1	319
Patients transferred/migrated to a different facility	(-) 35	284
Treatment outcome is pending	(-) 7	277
Treatment outcome should be one of cured, complete, death or treatment failure	(-) 1	276

**Table 2:** Control dataset: Selection criteria pathway.

Reason for inclusion/exclusion	#Patients Excluded	Patients retained
Patients notified in New Delhi between October 2019 and March 2020		9478
Patients who were engaged with the CfL application	(-) 333	9145
Patients notified at one of the 3 facilities	(-) 8407	738
Treatment outcome is pending	(-) 13	725
Treatment outcome should be one of cured, complete, death or treatment failure	(-) 12	713

### Model theory

**Fig 1:**
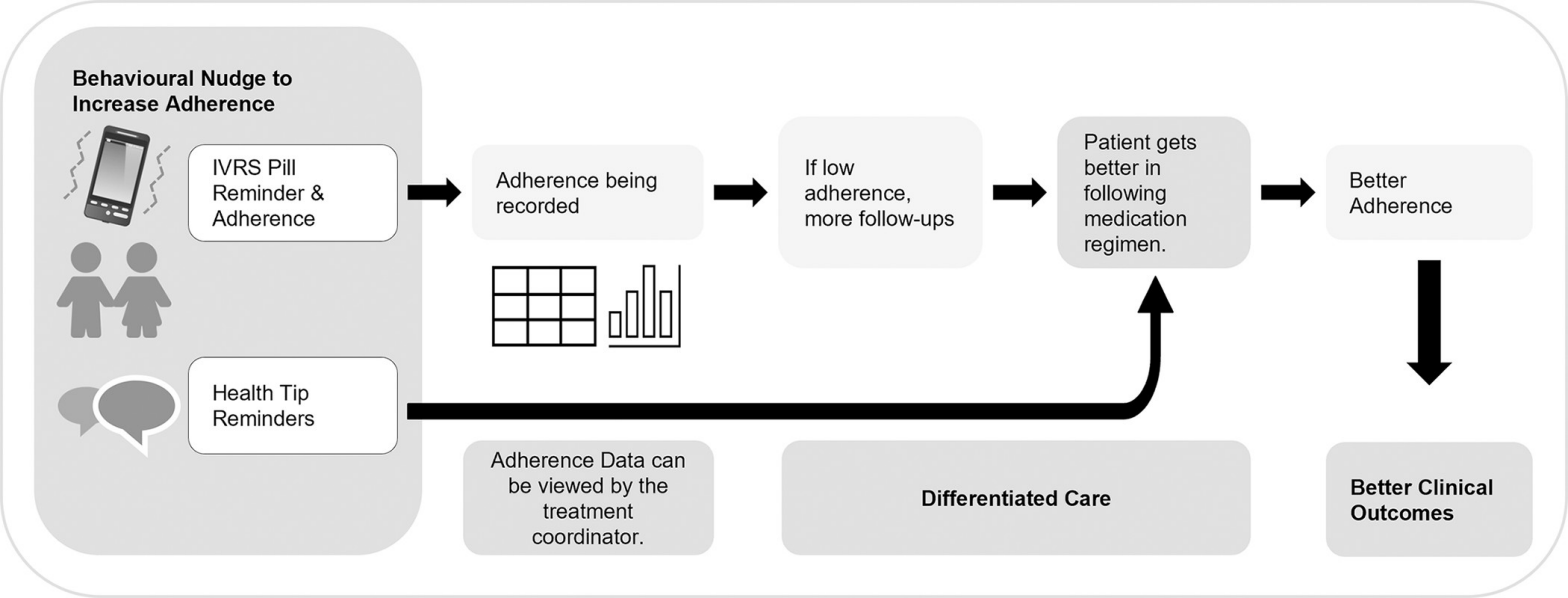
CfL Patient Care Cascade.

We applied multiple statistical models to understand how CfL engagement impacted patients and their treatment outcomes. Our findings suggest that the combination of features provided by the CfL application contributed to increased patient engagement with their treatment. Routine health tips, along with medication reminders, customized to be sent at a chosen time, based on patient’s preference reduced barriers to treatment adherence and encouraged patients to stay connected with their treatment coordinator. At the same time, treatment coordinators utilized the platform to monitor patients’ adherence, and increased follow-ups whenever the patients were found to lag behind in medication adherence. These factors contributed to improved treatment adherence, leading to better treatment outcomes (Fig 1). The study attempts to develop a precise estimate of the treatment effect of CfL on patients’ follow-ups and treatment outcomes using a combination of regression methods on a matched dataset built through propensity choice modelling.

### Outcomes of interest

The study has two primary outcomes, 1) patient follow-ups and 2) treatment outcomes. Patient follow-ups refer to the number of times a patient spoke to or met with a treatment coordinator, serving as a proxy for patient management. Treatment outcomes are analyzed as a binary variable in the study. Five outcomes are considered 1) treatment complete, 2) cured, 3) treatment failure, 4) death, and 5) lost to follow-up. The first two correspond to a successful outcome, and the last three correspond to an unsuccessful outcome. These outcomes are further described with clinical definitions in Table A in S1 Appendix [14].

### Propensity choice modelling

Effectively, all diagnosed TB patients who visited the three facilities during the pilot duration were offered to enroll in the CfL intervention, which implies that the data collected as part of this study was not randomized. This makes it difficult to access the average treatment effect (ATE) of the intervention on the outcomes of interest. While randomized experiments are considered the gold standard to understand the causal effect of a treatment, running such an experiment is often cost intensive and consists of multiple ethical issues (primarily concerning who receives the intervention), especially in studies related to welfare and healthcare treatment effects [15]. Hence, the practical challenges and ethical considerations in our context necessitated the use of propensity score matching (PSM). Several studies have acknowledged the usage of matching methods to infer causal insights from non-randomized observational data, specifically in the field of healthcare assessment [16,17]. It is documented that creating a dataset matched on choice attributes allows for estimating the average effect of the treatment as if it were a randomized experiment, which means if the access to CfL was randomly assigned to individual patients [18].

We utilized propensity score modelling to create a matched dataset comprising treated patients (enrolled in the CfL) and untreated patients (not enrolled), including data on potential confounders for each individual [16,19–22]. The propensity score refers to the conditional probability of a patient being enrolled in the pilot, given the values of all potential confounder [20]. This score was estimated for each patient in the full analytical dataset. These scores were then used to create comparable groups of people who were part of the pilot engagement (treated) and those who did not ever engage (untreated). The scores were adequate predictors of whether or not a patient enrolled in the pilot, as detailed in S2 Appendix. We acknowledge that PSM, despite its strengths, cannot fully eliminate biases inherent in observational studies. To mitigate these biases, we employed robust matching techniques and conducted extensive sensitivity analyses to ensure the robustness of our results. We identified pairs of observations with very similar propensity scores, but differing treatment status (CfL or not), and employed the full matching algorithm, first developed by Rosenbaum (1991) [23] and illustrated by Hansen (2004) [24]. It uses all available individuals in the data by grouping the individuals into a series of matched sets (subclasses), with each set containing at least one treated individual (who received the treatment of interest) and at least one comparison individual (who did not). Full matching optimally groups treated individuals with similar comparison individuals based on propensity scores. Treated individuals with many similar matches are grouped with more comparison individuals, while those with fewer matches are grouped with fewer comparison individuals. This flexibility is an advantage over the traditional k:1 matching, where each treated individual is matched with the same number of comparison individuals (k), regardless of match quality [25]. To counter bias, we adopted two measures. First, we used a calliper width of 0.2 for the age and district variables using nearest neighbour matching, ensuring matched pairs were no more than 0.2 standard deviations apart, as recommended by previous studies [26]. Second, we applied exact matching on four variables: 1) proportion of males, 2) proportion of extrapulmonary cases, 3) proportion of patients diagnosed using Xpert testing, and 4) access to free drugs.

### Statistical modelling

Using the matched dataset, we fit fixed-effects ordinary least squares (OLS) regression and fixed-effects logistic regression models to estimate the impact of CfL engagement on the number of follow-ups made with the patient and the likelihood of a successful treatment outcome, respectively. The methods were chosen for the simplicity of the inferences they provide, relative to other non-linear methods which might have been preferred if the dataset were sufficiently larger. As covariates in the OLS regression model, we fitted a series of models, sequentially including CfL engagement, diagnosing facility, sex, age category (0 to 5, 6 to 15, 16 to 19, 20 to 45, 46 to 65, and ≥ 66 years), TB type (pulmonary or extra pulmonary), whether Xpert diagnostics were used, access to free drugs, and the quarter in which the diagnosis was made.

The logistic regression model was fit to assess the likelihood of a patient receiving a successful outcome at the culmination of treatment. The same covariates went into the logistic regression model. The variables chosen for the models were carefully examined to ensure they add an incremental explanation to the model. Nevertheless, the model could have also benefitted from additional potential confounding variables (e.g., occupation of the patient, family income, type of household–joint or urban, caregiving responsibilities, TB history in self or family). The study relied on programmatic data collected as part of the JEET PPSA intervention in New Delhi. However, the current availability of variables, as displayed in the results, did allow for a more than satisfactory pair of matched control and intervention datasets. Diagnosing quarter was included in both the OLS and logistic regression models to control for seasonal program-related influences of patient care and adherence to treatment. Interaction effects of diagnosing facility and diagnosing quarter were considered in both models to account for the simultaneous effect of these two variables on the dependent variables [27].

To establish the linkage between follow-ups and treatment outcomes, a logistic model was fit with follow-ups as an additional dependent variable while including for the status of CfL engagement and other covariates.

### Sensitivity analysis

Multiple sensitivity analyses were conducted to understand the impact of CfL engagement on follow-ups and treatment outcomes. Results from these analyses are provided in Tables A and B in S4 Appendix. Our results were robust to specifications excluding cases where treatment outcome resulted in lost to follow-up or when we ran facility specific models. The results were also robust to alternative matching methods, wherein we utilized nearest neighbour matching along with exact matching for selected variables (Table C in S4 Appendix). The latter resulted in 204 matched pairs.

### Statistical software

Analysis was conducted in R 2022.07.01. The `MatchIt`package [28,29] was used for the propensity score matching procedure. The `broom`, `cobalt`, and `gtsummary`packages were used for visualizing fitted and residual values, generating balance plots from propensity choice modelling, and generating summary statistics, respectively. Packages used for data cleaning, preparing the analytical datasets, measuring skewness, and visualizing results were `dplyr`, `tidyr`, `moments`, and `ggplot2`.

### Results

Table 3 provides the demographic and clinical profiles of patients in the analytical dataset (989 patients) and matched dataset (944 patients). Table 4 further details this information, by segregating between the pilot engagement status. The matching process using propensity scores brought the propensity score difference between the treated and control group from 0.278 to 0, while balancing the mean difference between other covariates (Table C in S2 Appendix). Within the matched dataset, 56% patients were male, 20% were diagnosed using Xpert testing, 56% had extrapulmonary TB, and 88% of patients had a successful treatment outcome recorded (Table 3).

**Table 3:** Summary statistics for dataset before and after matching.

	Analytical Dataset	Matched dataset
Number of patients	989	944
**Males**	554 (56%)	530 (56%)
**Age Category**		
1. 0–5	9 (0.9%)	9 (1.0%)
2. 6–15	67 (6.8%)	65 (6.9%)
3. 16–19	91 (9.2%)	85 (9.0%)
4. 20–45	490 (50%)	477 (50%)
5. 46–65	246 (25%)	226 (24%)
6. >65	86 (8.7%)	82 (8.7%)
**Age**		
Median (IQR)	35 (23, 53)	35 (23, 53)
Mean	38	38
**Free drugs**	117 (12%)	91 (9.6%)
**Xpert Testing**	221 (22%)	185 (20%)
**Extra Pulmonary**	545 (55%)	528 (56%)
**Follow Ups**	13 (6, 18)	13 (6, 18)
Median	13 (6, 18)	13 (6, 18)
Mean	12	12
Unknown	215	199
**Facility**		
sir ganga ram	529 (53%)	519 (55%)
st stephens	282 (29%)	256 (27%)
vinod karhana	178 (18%)	169 (18%)
**Treatment Outcome**		
complete	867 (88%)	829 (88%)
cured	3 (0.3%)	3 (0.3%)
died	91 (9.2%)	85 (9.0%)
failure	1 (0.1%)	1 (0.1%)
lost	27 (2.7%)	26 (2.8%)
**Successful Treatment Outcome**	870 (88%)	833 (88%)

***Note***: *1) The table showcases the numbers segregated by CfL status and within group percentages for them; 2) the p value for testing difference of means in groups with and without CfL; 3) n (%); Median (IQR) is given for continuous variables (age & follow ups)*

**Table 4:** Summary statistics for dataset before and after matching; segregated by the CfL pilot engagement status.

	Analytical Dataset			Matched dataset		
	(N = 989)			(N = 944)		
Pilot engagement	no CfL	CfL	*p-value*	no CfL	CfL	*p-value*
**Number of patients**	713	276		694	250	
**Males**	406 (57%)	148 (54%)	0.3	398 (57%)	132 (53%)	0.2
**Age Category**						
1. 0–5	6 (0.8%)	3 (1.1%)		6 (0.9%)	3 (1.2%)	
2. 6–15	45 (6.3%)	22 (8.0%)		45 (6.5%)	20 (8.0%)	
3. 16–19	51 (7.2%)	40 (14%)		51 (7.3%)	34 (14%)	
4. 20–45	336 (47%)	154 (56%)		334 (48%)	143 (57%)	
5. 46–65	200 (28%)	46 (17%)		186 (27%)	40 (16%)	
6. >65	75 (11%)	11 (4.0%)		72 (10%)	10 (4.0%)	
**Age**			<0.001			<0.001
Median (IQR)	38 (24, 56)	30 (20, 42)		37 (24, 56)	30 (20, 42)	
Mean	40	33		40	33	
**Free drugs**	48 (6.7%)	69 (25%)	<0.001	41 (5.9%)	50 (20%)	<0.001
**Xpert Testing**	105 (15%)	116 (42%)	<0.001	88 (13%)	97 (39%)	<0.001
**Extra Pulmonary**	408 (57%)	137 (50%)	0.031	402 (58%)	126 (50%)	0.040
**Follow Ups**						<0.001
Unknown	185	30		173	26	
Median (IQR)	11 (4, 16)	18 (13, 20)	<0.001	11 (4, 16)	18 (13, 20)	
Mean	10	16		10	16	
**Facility**			<0.001			<0.001
sir ganga ram	454 (64%)	75 (27%)		447 (64%)	72 (29%)	
st stephens	153 (21%)	129 (47%)		143 (21%)	113 (45%)	
vinod karhana	106 (15%)	72 (26%)		104 (15%)	65 (26%)	
**Successful treatment outcome**	608 (85%)	262 (95%)	<0.001	595 (86%)	237 (95%)	<0.001

***Notes***: *1) The table showcases the numbers segregated by CfL status*, *and within group percentages for them; 2) For binary/character variables*, *values represent the number of patients enrolled*, *and value in parentheses represents share or %; 3) For continuous values*, *the number represents the median*, *and the values in parentheses represents the Interquartile Range; 4) Pearson’s Chi-squared test and Kruskal-Wallis rank sum test is conducted for p value*

### Follow-ups with patients

**Fig 2:**
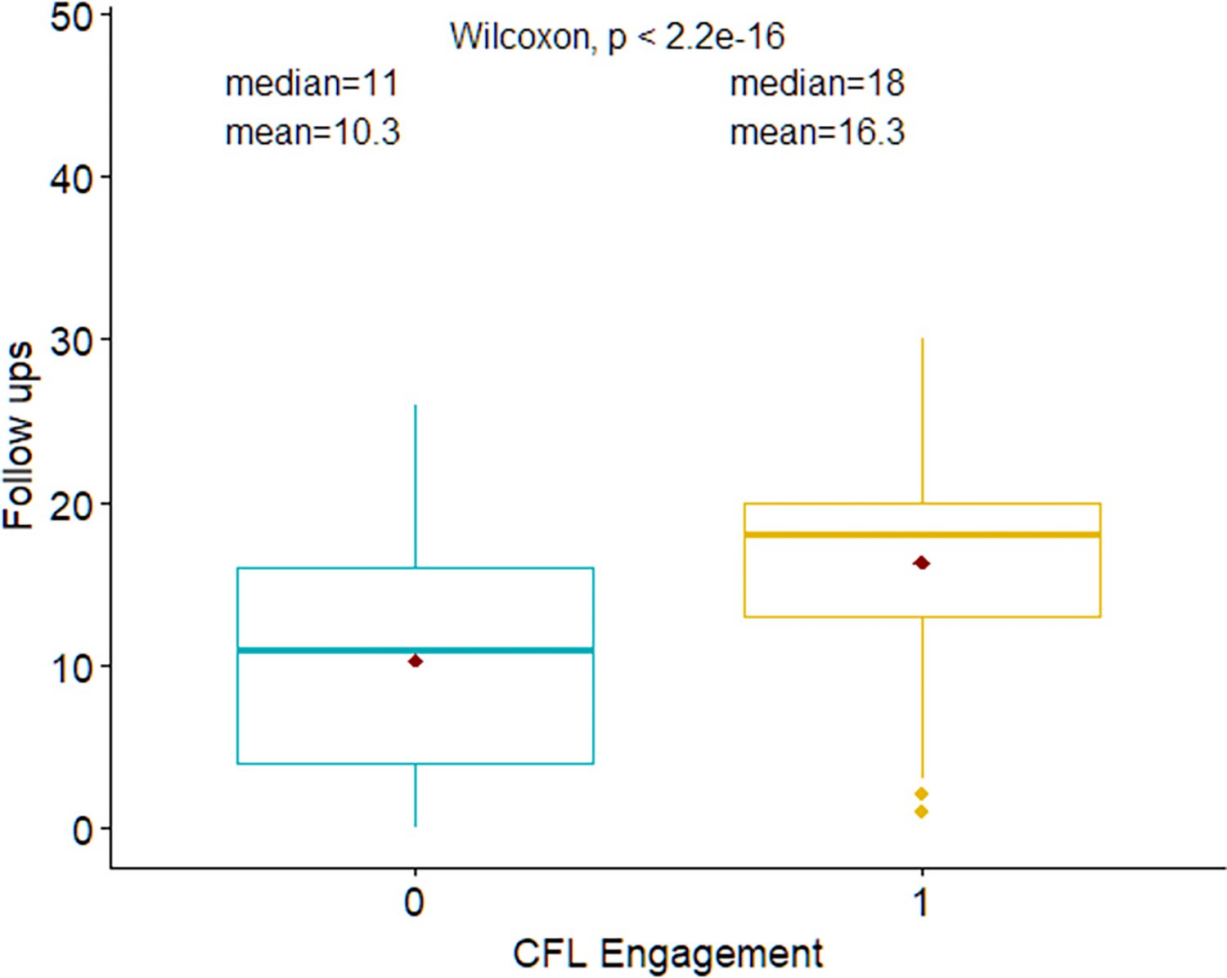
Box plot showing the difference in follow ups made with patients; based on whether they were a part of the CfL pilot; Matched Dataset; N = 944. *Note*: The diamond dot represents mean, and the box boundary represents the inter-quartile distribution.

Within the matched dataset, patients with CfL engagement received more follow-ups from treatment coordinators (Mean = 16.3, Median (IQR): 18 (13,20)) compared to those who were not engaged with the pilot (Mean = 10.3, Median (IQR): 11 (4, 16)). These differences are illustrated in Fig 2 as a box plot distribution. We fit a series of five regression models that progressively added patient-level covariates, fixed-effects for facility, patient level covariates, fixed-effects for quarter of diagnosis, and an interaction between facility and quarter of diagnosis (Table 5).

**Table 5:** OLS regression results showing impact of the pilot CfL engagement on number of follow-ups with patients using matched dataset; N = 745.

	Model A	Model B	Model C	Model D	Model E
CfL Engagement	5.995	6.128	6.131	5.943	6.417
95% C.I.	(4.986, 7.003)	(5.070, 7.185)	(4.963, 7.298)	(4.768, 7.119)	(5.295, 7.540)
+ Facility fixed effects		Yes	Yes	Yes	Yes
+ Additional Covariates			Yes	Yes	Yes
+ Diagnosing Quarter fixed effects				Yes	Yes
+ Facility by Diagnosing quarter interaction					Yes
Observations	745	745	745	745	745
R^2^	0.150	0.154	0.176	0.180	0.216
Adjusted R^2^	0.148	0.151	0.162	0.166	0.200
Residual Std. Error	6.561 (df = 743)	6.552 (df = 741)	6.507 (df = 732)	6.494 (df = 731)	6.359 (df = 729)
F Statistic	130.748 (df = 1; 743)	45.098 (df = 3; 741)	13.026 (df = 12; 732)	12.374 (df = 13; 731)	13.417 (df = 15; 729)
	(df = 1; 745)	(df = 3; 743)	(df = 12; 734)	(df = 13; 733)	(df = 15; 731)

Note: a) 95% C.I. based on robust standard errors; b) All models were fitted on matched dataset; c) *p<0.1, **p<0.05, ***p<0.01

**Fig 3:**
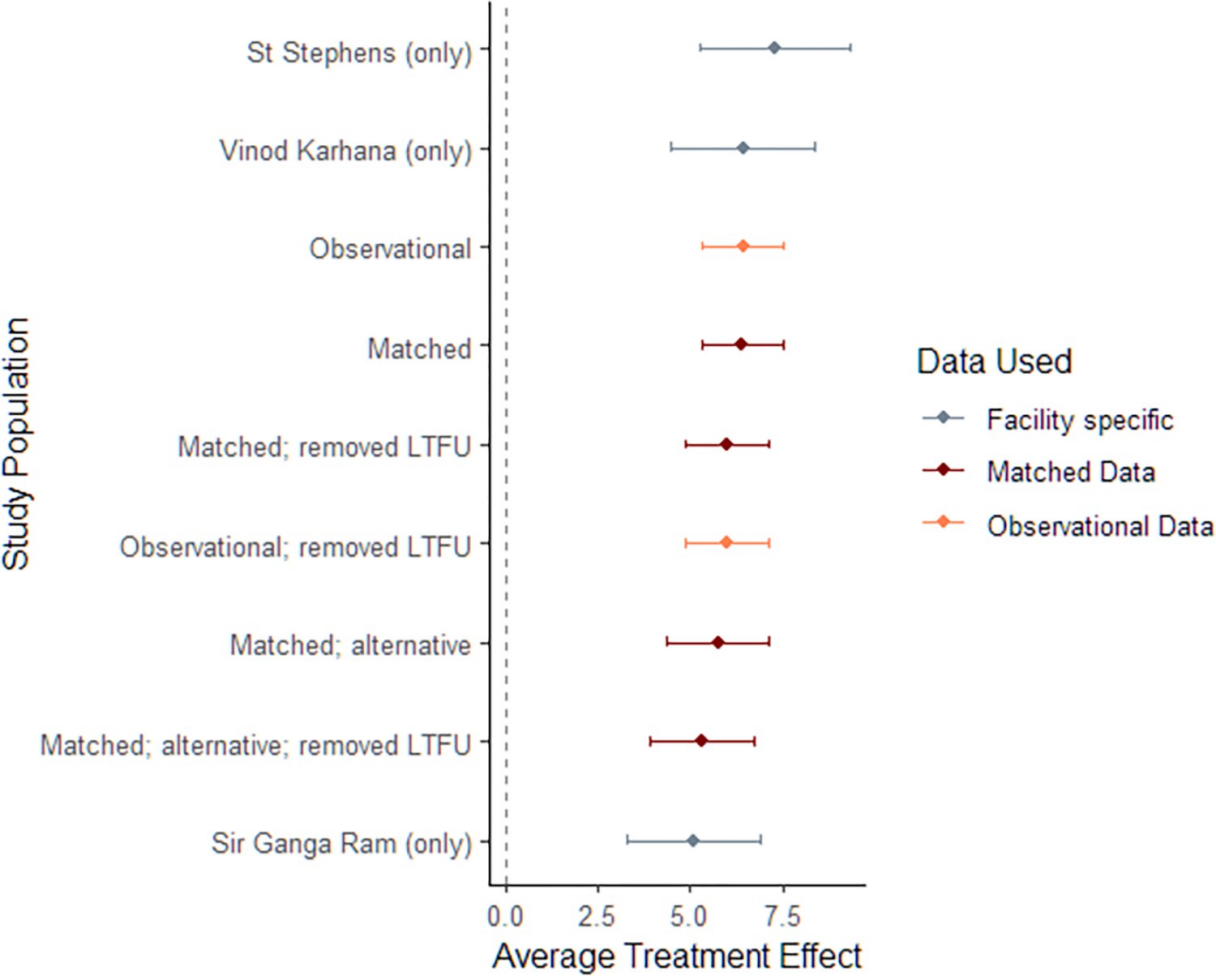
Forest Plot for OLS Regression; Impact of CfL on follow Ups, Effect Size by alternative sub-population and matching specification. *Note*: *1) LTFU refers to Lost to follow up; 2) Dotted line at X = 0 helps in visualizing the sub populations which reveal a significant impact (or not) of CfL on follow ups outcomes; 3) All facility specific models are fitted on the matched dataset; 4) Matched (alternative) refers to an alternative matching specification detailed in Table C in* S4 *Appendix*.

All five models revealed statistically significant effect of the pilot engagement on follow-ups, with Model E controlling for all available potential confounders and including an interaction term on diagnosing facility and quarter. An average treatment effect (ATE) of 6.4 additional follow-ups (95% C.I. = 5.295 to 7.540) was found for patients enrolled in the pilot relative to those who were not. This corresponds to a 62% increase in mean follow-ups (58% increase when comparing median values). Fig 3 demonstrates the robustness of our study through a forest plot, presenting results across different sub-population groups and various matching specifications.

### Treatment outcomes

**Fig 4:**
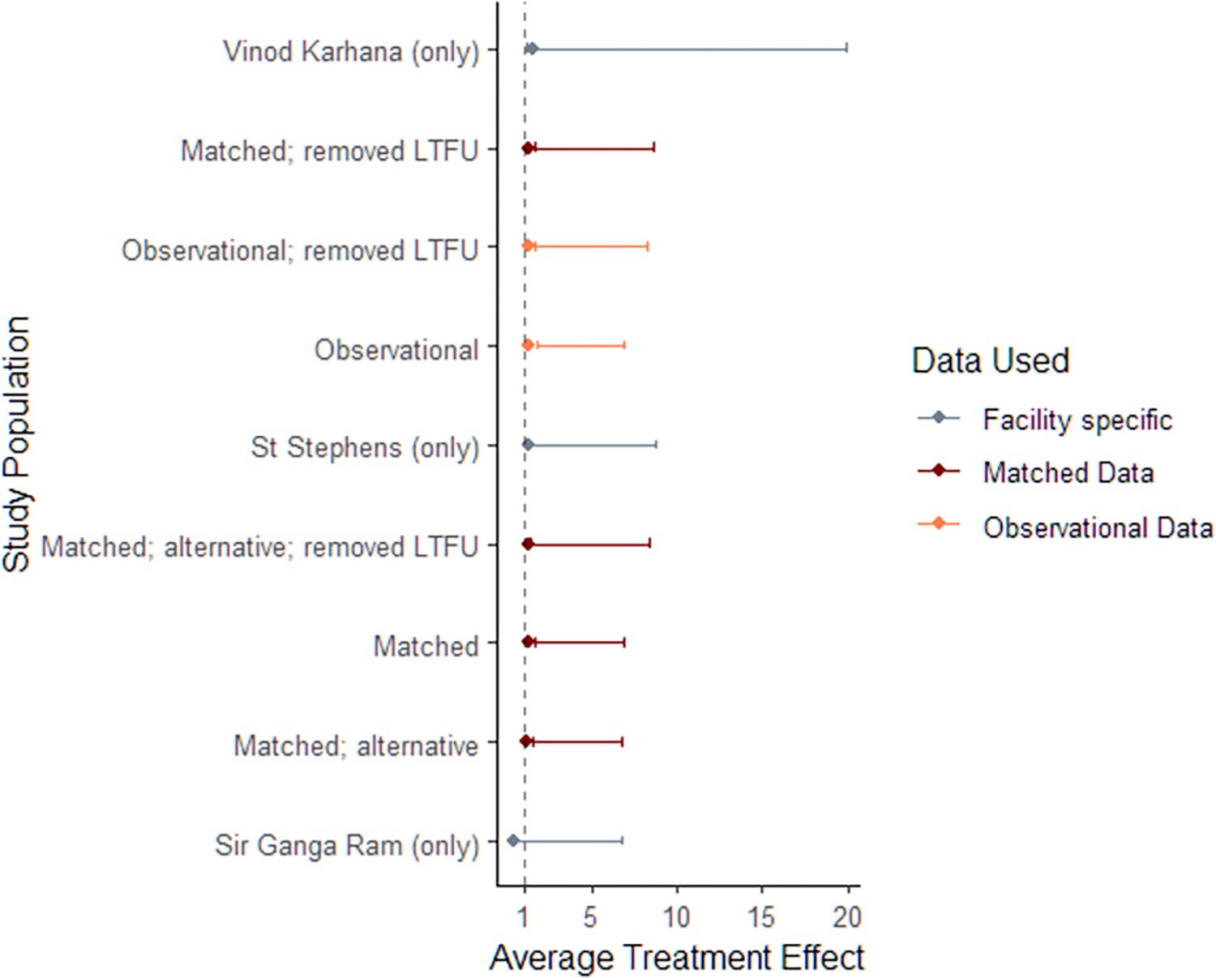
Forest Plot for Logistic Regression; Impact of CfL on Treatment Outcomes, Effect Size by alternative sub-population and matching specification. *Note*: *1) OR Ratios are displayed along with 95% C*.*I*.*; 2) Dotted line at X = 1 illustrates the sub-populations which reveal a significant impact of CfL on treatment outcomes (to the right) or not (to the left) 3) LTFU refers to lost to follow-up; 4) All facility specific models are fitted on the matched dataset; 5) Matched (alternative) refers to an alternative matching specification detailed in Table C in* S4 *Appendix*.

A series of fixed-effects logistic regression models (Table 6) revealed a statistically significant greater likelihood of a successful treatment outcome for patients enrolled in the CfL pilot, compared to those who were not. Model E, which includes controls for all available covariates, estimates 242% higher odds (OR = 3.415; 95% C.I. [1.701 to 6.857]) of a successful outcome for patients who were enrolled in the pilot relative to others. Fig 4 showcases the robustness of these results through a forest plot, presenting results across various sub-population groups and various matching specifications.

**Table 6:** Logistic regression results showing impact of the pilot CfL engagement on treatment outcomes using matched dataset; N = 944.

	Model A	Model B	Model C	Model D	Model E
CfL Engagement	3.033***	4.631***	3.54***	3.589***	3.415***
95% C.I.	(1.668,5.517)	(2.483,8.635)	(1.776,7.056)	(1.809,7.12)	(1.701,6.857)
+ Facility fixed effects		Yes	Yes	Yes	Yes
+ Additional Covariates			Yes	Yes	Yes
+ Diagnosing Quarter fixed effects				Yes	Yes
+ Facility by Diagnosing quarter interaction					Yes
Observations	944	944	944	944	944
Log Likelihood	-335.455	-319.634	-288.460	-288.411	-286.184
Akaike Inf. Crit.	674.911	647.268	602.920	604.823	604.368

Note: a) 95% C.I. based on robust standard errors; b) All models were fitted on matched dataset; c) *p<0.1, **p<0.05, ***p<0.01; d) Treatment outcome is equal to success (= 1) if treatment is completed or cured. Unsuccessful outcome refers to lost to follow up, death or treatment failure

### Link between CfL, follow-ups, and treatment outcomes

Including follow-ups as a covariate in the logistic model reduces the size and significance of the coefficient on CfL (Table 7). It also reveals a statistically significant coefficient on the follow-ups, estimating a 24% increased likelihood of a successful outcome for every additional follow up with the patient. Results from this specification (Table 7), along with the model revealing a significant impact of CfL drugs on follow-ups (Table 5), lead us to conclude that the CfL engagement results in better treatment outcomes, primarily through its impact on the number of follow-ups made with the patient.

**Table 7:** Logistic regression results showing impact of CfL on treatment outcomes using matched dataset; N = 745; including follow ups as a covariate.

	Model A (Odds Ratio)	95% C.I.
CfL	0.444	(0.181, 1.088)
Follow ups	1.242***	(1.174, 1.313)
All Covariates	Yes	
Diagnosing Quarter FE	Yes	
District FE	No	
Treatment Coordinator FE	Yes	
Observations	745	
Log Likelihood	-156.047	
Akaike Inf. Crit.	346.093	

**Note**: a) 95% C.I. based on robust standard errors; b) The model was fitted on the matched dataset; c) *p<0.1, **p<0.05, ***p<0.01

## Discussion

To our knowledge, this is the first study assessing the impact of a DAT intervention on TB patients seeking care in the Indian private sector using a quasi-experimental approach. Our findings illustrate that patients who used the CfL application had a significantly higher likelihood of completing their treatment successfully, likely caused by an increase in follow-ups associated with the usage of the application. The results remain robust after employing propensity score matching methods and a series of sensitivity analysis. Additionally, the study not only adds to the limited quantitative evidence on the impact of DATs but also highlights the practical implementation challenges and potential for these technologies to support healthcare workers and improve patient engagement in TB care. We find that such mobile technologies, when combined with human-centric interventions in terms of patient follow-ups, could lead to highly meaningful improvements in treatment outcomes. While DATs) have been increasingly used for improving patient behaviours across the globe, their particular usage in India has remained underutilized, in part due to a lack of proper regulation and implementation [30]. Additionally, in-person or telephonic follow-ups, though a gold standard to improve all aspects of patient management, can be burdensome for developing countries such as India because of high patient load, static workforce and high number of patients living in rural and remote areas [31]. A study in Uganda illustrated the role played by such DATs where face to face counselling and social support is expensive because of a lack of financial resources and difficulties in transportation [32]. Wider usage of digital applications has the potential to mitigate these challenges [3]. Our study finds that those who enrolled in the intervention received 6.4 additional follow-ups, resulting in a more than 60% increase in engagement with the treatment coordinator. This heightened engagement is significantly linked to a 242% higher likelihood of treatment completion, which is crucial for preventing TB relapse in diagnosed individuals. Studies have highlighted the role of effective communication through mHealth technologies in India, which could bridge the gap between patient and medical staff interaction [33]. Treatment for TB is particularly complex and long drawn, which further intensifies the need for novel methods to ensure patients adhere to the treatment through various challenges [34]. A modelling analysis estimates that such DATs, if employed in the public sector alone, can potentially reduce TB incidence by 7.3% over a course of 10 years, and by 16% if also deployed in the private sector, albeit under idealized settings [9]. Another study conducted in Bengaluru, India highlighted the role of an mHealth application (Kill TB) in using reminders to improve patient adherence [35].

Previous examples of digital interventions to manage adherence include 99DOTS [36], Video Directly Observed therapy (VDOT) or Video Observed therapy (VOT) and Event Monitoring device for medication support (EMM) [37]. The current landscape continues to evolve, with ongoing iterations of these devices and solutions being piloted to better understand their use cases. Recent evaluations of the Medical Event Reminder Monitor (MERM) box [10] and TMEAD [38], both applications of EMM, have shown favourable outcomes among patients using these solutions, albeit stating challenges with respect to actual usability [10,38–41]. Previous studies have recommended these DATs to be used to support a larger patient management system, offering options like differentiated counselling or switching to DOT in cases of non-adherence [42–44]. While all these interventions included a dashboard solution to enable patient monitoring by healthcare workers, most evaluative studies have not specifically assessed its impact [2,45]. In our study, we observed that patients enrolled in the CfL intervention received a higher number of follow-ups. Earlier studies have highlighted the role of enabling differentiated care [46] and the importance of human interactions in improving the effectiveness of DATs [47–49]. A previous systematic review talks about using such DATs to enable differentiated care, and more intensive face-to-face engagement as and when required [3]. Given the mixed evidence on the impact of DATs, our findings suggest that digital technologies may have limited impact if used in isolation. Recent studies have highlighted both patient and provider concerns in deploying DATs, particularly those utilizing artificial intelligence (AI) for medication and counselling support [50–52]. Patients are found to prefer systems where healthcare workers oversee and validate the recommendations provided by these technologies, ensuring that human judgment complements AI mechanisms [52]. On the other hand, providers are found to have concerns about maintaining patient privacy, ensuring correct prescriptions, and integrating these systems into workflows [50]. Our study demonstrates that technology can support healthcare workers, enhancing treatment outcomes and patient engagement while emphasizing the importance of human interaction in healthcare delivery. A recently published cluster-randomized trial, conducted in China, aimed to evaluate the impact of DATs on TB treatment outcomes. Despite the significant positive impact on medication adherence, the study found no effect on unfavourable outcomes such as loss to follow-up, recurrence, death, and treatment failure [46]. The study highlighted significant issue of patient retention in treatment programs and the importance of managing follow-up effectively. Our intervention combined the availability of the CfL application with the support of treatment coordinators whose follow-ups were guided by the recorded adherence data in the application. This combined approach likely explains the positive impact on treatment outcomes observed in our analysis. In contrast, the cluster-randomized trial in China emphasizes the complexity of improving TB treatment outcomes through digital interventions alone and underscores the importance of timely review of adherence data and the implementation of differentiated care strategies [46]. These findings reinforce that medication non-adherence is a multifaceted issue influenced by various factors [53,54]. Therefore, tailored interventions are essential to achieve positive impact from any technological solution [3].

## Conclusions

The results from this analysis are significant in illustrating the impact of the CfL solution in improving the ability of healthcare workers to counsel patients effectively, while simultaneously improving a patient’s engagement with their treatment by offering a combination of services such as health tips delivery, drug medication reminders, and clinic visit reminders. Our findings underscore the need for further research to validate the long-term effects of such interventions and explore their scalability and integration into broader healthcare systems. Digital interventions such as these serve as a low-cost method to improve patient behaviours with respect to continuing treatment, especially among populations lacking access to clinics, such as those in remote rural areas where transportation cost pose a significant barrier to healthcare access. They also help reduce stigma and generate awareness among patients and caregivers, potentially improving patient attitudes to treatment and care. Such interventions also have the potential to reduce the psychological burden borne by healthcare workers in resource-constrained settings. Future large-scale deployments of such technologies need to consider the importance of a multifaceted intervention, combining the elements of technology and human-centred approach to optimize patient care and improve treatment outcomes.

## Limitations

Our analysis strongly suggests the impact of the CfL intervention, but there are several limitations that warrant further research. First, although we employed robust matching techniques and conducted extensive sensitivity analyses to ensure the robustness of our results, further validation through RCTs or other rigorous experimental designs is needed to confirm our findings. Second, our study was conducted in three facilities within New Delhi, including a sample size of fewer than 1000 individuals, limiting the generalizability of the findings. Implementing the application across a larger and more diverse population, with additional socio-demographic data, would enable a more comprehensive assessment of DATs and provide deeper insights into patient adherence behaviours. Third, our analysis does not investigate the specific implementation challenges witnessed by the program team, which would be essential to initiating a scale up of the same across a wider geography and/ or a greater number of facilities. Some of the impediments noted by the program staff included a) disruptions in internet/telephone-network at the home location of patients, and b) patients complaining about redundant or repetitive content in health tip deliveries, and c) lengthy process to record their adherence into the system. Despite these challenges, the CfL application was broadly flexible to individual patients’ needs. For instance, patients or caregivers could adjust the frequency of reminders, set preferred times for notifications, and select the topics on which they wanted nudges (e.g., pill reminders, doctor visit reminders, adherence reports, nutrition, and potential side effects). However, quickly addressing similar challenges within a built-in software solution can be cumbersome, especially when deployed across a larger patient group. Notwithstanding the plausible challenges, several of the commonly found difficulties with using such solutions (application crash, data recording errors, erroneous or harmful information in terms of health tips) [55] were not reported from patients or the program staff using this application. Addressing these challenges in future implementations will be crucial for optimizing the effectiveness and user experience of digital adherence technologies. Fourth, our analysis does not delve into the specifics of why we have more “unknown” follow-ups for patients who do not enroll in the intervention. We suspect two primary reasons for this–a) lack of consent / interest from the patients’ side to speak with a treatment coordinator, and b) an increased motivation on the part of the treatment coordinator to speak with patients enrolled in the intervention. It is difficult to assess which of the two reasons dominated, if at all, and how this might be inferred from the context of scaling up similar interventions. Large scale interventions based on this combined intervention approach will benefit from understanding the motivation factors of treatment coordinators to follow-up with certain patients over others. Fifth, while the results are accompanied by a facility-level sensitivity analysis, our research does not attempt to find reasons for heterogeneity in results obtained or delve into the specifics of implementation in these different facilities. It is worth noting that Vinod Karhana is a relatively smaller facility, with respect to the number of patients catered to, when compared with Sir Ganga Ram hospital and St. Stephens hospital. Both Sir Ganga Ram and St. Stephens are situated in central Delhi, making it relatively easier for patients to access them by various forms of public transportation. A more detailed, and perhaps qualitative narrative of how such interventions might impact patients visiting such diverse facilities is warranted. The same can help inform differential enrolment and investment strategies, which can be more efficient in utilizing such DATs for TB, as well as other diseases involving long and complicated treatment regimens. Sixth, we focused on treatment outcomes rather than adherence data, as adherence is self-reported and prone to bias. Our outcomes-based approach evaluated the impact on follow-ups and successful treatment completions. However, researching medication adherence could reveal more specific barriers to patient engagement and the successful deployment of such interventions. Seventh, we use a derived dichotomous outcome variable to understand the impact of the intervention on treatment outcomes. Here, unsuccessful outcomes include treatment failure, death, and lost to follow-up, and each of these outcomes may have their own risk profiles. All patients who had a treatment interruption greater than one month in duration are considered as being lost to follow-up. However, it cannot be determined if patients continued the treatment later, and if so, whether they were able to complete the treatment with a positive outcome. Hence, including lost to follow-up has the potential to bias these results. Some previous studies have not included lost to follow-up in their analyses for similar reasons [56]. However, we remain conservative and followed the baseline criteria of including patients who were under the active management of a treatment coordinator, and had their outcomes reported at least a month after the date of diagnosis. Additionally, in our analysis, lost to follow-up makes up 2.7% and 2.8% of our analytical and matched datasets, respectively. Including lost to follow-up in analysis where these cases make less than <5% of the overall population generally leads to little bias [57]. Sensitivity analysis excluding patients with lost to follow-up as a treatment outcome (S4 Appendix) supported the primary findings with high statistical significance. Regardless, further research is warranted to fully understand the differential risk profile of private sector TB patients, including the drivers of lost to follow-up and treatment failure. Lastly, most patients reported treatment completion based on provider declarations, but the metric of successful treatment completion has limitations due to low cure rates from lack of smear testing in the private sector.

## Supporting information

S1 Appendix Distinct treatment outcomes, details of what is included or not, along with definitions. (DOCX)

S2 Appendix Propensity Scores Modeling, details of the modeling process. (DOCX)

S3 Appendix Regression Results, full model results. (DOCX)

S4 Appendix Sensitivity Analysis, details of distinct models fitted in order to check robustness of results. (DOCX)
