# Supplementary material for: Impact of a pilot mHealth intervention on treatment outcomes of TB patients seeking care in the private sector using Propensity Scores Matching—Evidence collated from New Delhi, India
Source: PLOS Digit Health. 2024 Sep 11;3(9):e0000421. doi: 10.1371/journal.pdig.0000421 (PMC11389929; doi:10.1371/journal.pdig.0000421)
Supplement: S1 Appendix — (DOCX) [file pdig.0000421.s001.docx]

# Appendix 1: Definition of treatment outcomes

We give below the definition of different treatment outcomes, as gathered by the Technical and Operational Guidelines for TB Control in India [1]*.*

**Table A. Definitions of treatment outcomes for drug susceptible TB patients.**

| **Treatment outcome** | **Definition** | **Study outcome** | **Considered for the study** |
| --- | --- | --- | --- |
| Cured | Microbiologically confirmed TB patients at the beginning of treatment who was smear or culture negative at the end of the complete treatment | Successful treatment | Yes |
| Treatment complete | Completed treatment without evidence of failure or clinical deterioration but with no record that the smear or culture results of biological specimen in the last month of treatment was negative |  |  |
| Lost to follow up | Treatment was interrupted for one consecutive month or more | Unsuccessful treatment |  |
| Died | Died during the course of anti-TB treatment |  |  |
| Treatment failure | Biological specimen is positive by smear or culture at end of treatment |  |  |
| Not evaluated | Patients for whom no treatment outcome is assigned; also includes former transfer outs | Other outcomes (not considered) | No |
| Treatment regimen changed | A TB patient who is on first line regimen and has been diagnosed as having DR TB and switched to drug resistant TB regimen prior to being declared as failed |  |  |
| Wrongly diagnosed | A patient who is wrongly diagnosed of TB |  |  |
| Transferred | A patient who has transferred to another facility or state, prior to the outcome being declared |  |  |

# References

1. Central TB Division. Treatment Outcomes for drug susceptible TB patients. In: Technical and Operational Guidelines for TB Control in India. 2016. p. 65.
