# Supplementary material for: Impact of a pilot mHealth intervention on treatment outcomes of TB patients seeking care in the private sector using Propensity Scores Matching—Evidence collated from New Delhi, India
Source: PLOS Digit Health. 2024 Sep 11;3(9):e0000421. doi: 10.1371/journal.pdig.0000421 (PMC11389929; doi:10.1371/journal.pdig.0000421)
Supplement: S2 Appendix — (DOCX) [file pdig.0000421.s002.docx]

# **Appendix** **2 –Propensity Scores Modeling**

We utilized propensity score modelling [1–3] to create a matched dataset comprised of patients who registered for the intervention (CfL) and those who did not receive the intervention. We use a logistic regression model, including all potential confounders. The exact model results from the same are given in Tables A and B. The accuracy of the model so obtained is 77% with a specificity rate of 19%. We also illustrate the results graphically in Fig A, which visually displays the distribution of propensity scores, separated by whether or not the patient was enrolled in the CfL program.

## Propensity score using logistic regression

**Table A. Estimating propensity score using logistic regression; N = 989**

| **Dependent Variable: CfL Intervention** | |
| --- | --- |
| male | 0.955 (0.622, 1.288) |
| Age: 6-15 | 0.612 (-1.036, 2.259) |
| Age: 16-19 | 0.817 (-0.794, 2.428) |
| Age: 20-45 | 0.735 (-0.817, 2.288) |
| Age: 46-65 | 0.338 (-1.248, 1.924) |
| Age: > 65 | 0.240 (-1.466, 1.945) |
| Xpert Testing | 2.539 (2.111, 2.968) |
| Free drugs | 11.113 (10.594, 11.632) |
| Extra Pulmonary status | 1.027 (0.675, 1.379) |
| Facility: St Stephens | 6.075 (5.585, 6.564) |
| Facility: Vinod Karhana | 8.295 (7.795, 8.794) |
| Diag Qtr: 2020 Q1 | 2.708 (2.370, 3.047) |
| Constant | 0.069 (-1.539, 1.678) |
| Observations | 989 |
| Log Likelihood | -440.605 |
| Akaike Inf. Crit. | 907.209 |
| Note: a) 95% C.I. based on robust standard errors; b) *p<0.1; **p<0.05; ***p<0.01; c) 95% C.I. displayed alongside results | |

## Mean Propensity scores – by CfL intervention

**Table B. Propensity Score estimated by whether or not a patient was registered on CfL intervention**

| **enrolled in CfL** | **N** | **Mean score** | **Median score** |
| --- | --- | --- | --- |
| **No** | 713 | 0.20 | 0.12 |
| **Yes** | 276 | 0.48 | 0.49 |

**Fig A. Histograms of the estimated propensity scores by treatment status**


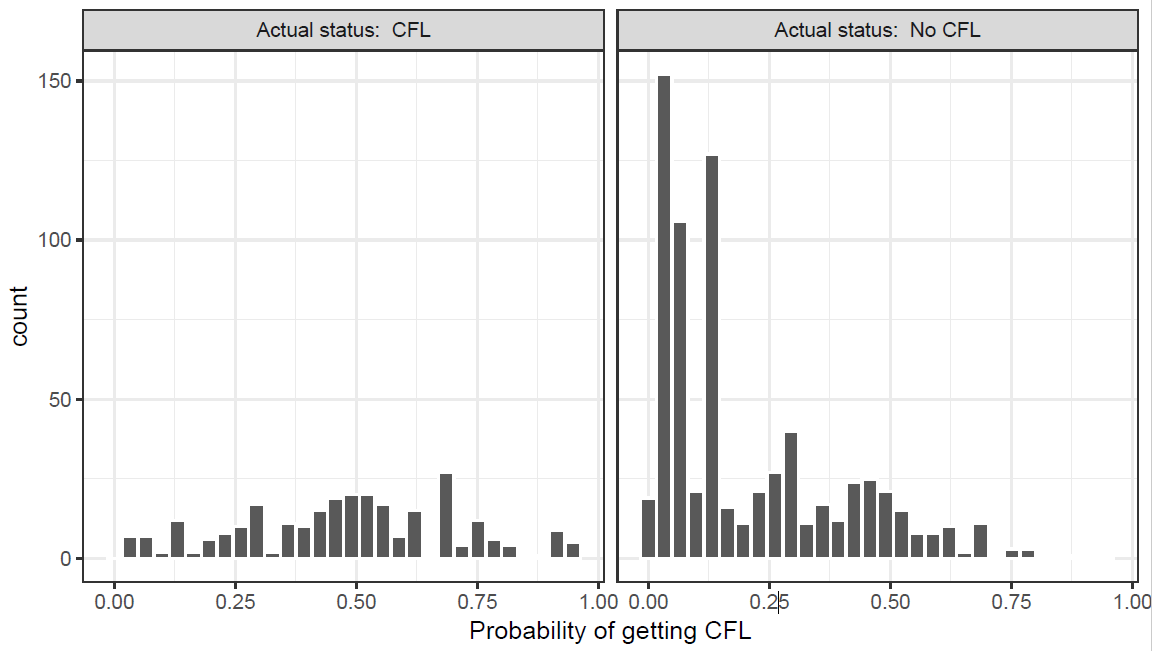


## Summary of Balance Table – before and after matching

In propensity score modelling, the balance table summarizes the differences in covariates between the treatment and control groups before and after matching. This balance table helps in verifying that the propensity score matching has successfully created comparable groups, minimizing bias in the estimation of treatment effects. We explain the four variables depicted in Table C:

1. Mean Values: The average values of each covariate for both groups before and after matching,
2. Difference in Mean values: Difference in mean values for intervention (Treated) and control groups,
3. Standardized Mean Differences (SMDs): This metric measures the effect size, with values less than 0.1 indicating good balance; and,
4. Variance Ratios: Ratios of variances of covariates between groups, with values close to 1 suggesting balanced variances

**Table C. Summary of balance for the dataset, before and after matching.**

|  | **Analytical Dataset** | | | | | **Matched Dataset** | | | | |
| --- | --- | --- | --- | --- | --- | --- | --- | --- | --- | --- |
|  | **Means Treated** | **Means Control** | **diff** | **Std. Mean Diff.** | **Var. Ratio** | **Means Treated** | **Means Control** | **diff** | **Std. Mean Diff.** | **Var. Ratio** |
| Diff | 0.480 | 0.201 | 0.278 | 1.247 | 1.366 | 0.444 | 0.444 | 0.000 | 0.000 | 0.996 |
| Age: 0-5 | 0.011 | 0.008 | 0.002 | 0.024 |  | 0.012 | 0.011 | 0.001 | 0.010 |  |
| Age: 6-15 | 0.080 | 0.063 | 0.017 | 0.061 |  | 0.080 | 0.057 | 0.023 | 0.085 |  |
| Age: 16-19 | 0.145 | 0.072 | 0.073 | 0.209 |  | 0.136 | 0.125 | 0.011 | 0.030 |  |
| Age: 20-45 | 0.558 | 0.471 | 0.087 | 0.175 |  | 0.572 | 0.564 | 0.008 | 0.016 |  |
| Age: 46-65 | 0.167 | 0.281 | -0.114 | -0.305 |  | 0.160 | 0.174 | -0.014 | -0.038 |  |
| Age: >65 | 0.040 | 0.105 | -0.065 | -0.334 |  | 0.040 | 0.069 | -0.029 | -0.147 |  |
| Male | 0.536 | 0.569 | -0.033 | -0.067 |  | 0.528 | 0.528 | 0.000 | 0.000 |  |
| Xpert Testing | 0.420 | 0.147 | 0.273 | 0.553 |  | 0.388 | 0.388 | 0.000 | 0.000 |  |
| Free drugs | 0.250 | 0.067 | 0.183 | 0.422 |  | 0.200 | 0.200 | 0.000 | 0.000 |  |
| Facility: sir ganga ram | 0.272 | 0.637 | -0.365 | -0.821 |  | 0.288 | 0.271 | 0.017 | 0.038 |  |
| Facility: st stephens | 0.467 | 0.215 | 0.253 | 0.507 |  | 0.452 | 0.462 | -0.010 | -0.020 |  |
| Facility: vinod karhana | 0.261 | 0.149 | 0.112 | 0.256 |  | 0.260 | 0.267 | -0.007 | -0.016 |  |
| Diag Qtr: 2019 Q4 | 0.355 | 0.516 | -0.161 | -0.337 |  | 0.380 | 0.374 | 0.006 | 0.013 |  |
| Diag Qtr: 2020 Q1 | 0.645 | 0.484 | 0.161 | 0.337 |  | 0.620 | 0.626 | -0.006 | -0.013 |  |
| Extra Pulmonary | 0.496 | 0.572 | -0.076 | -0.152 |  | 0.504 | 0.504 | 0.000 | 0.000 |  |

## Histogram and Love Plot – displaying summary of balance visually, before and after matching

Figs B and C display the same information visually, by way of a histogram and Love Plot, respectively [4]. While Fig B shows the distribution of propensity scores for both treatment and control groups, Fig C highlights the standardized mean differences before and after the matching.

**Fig B. Propensity scores, before and after the matching, in the treated (CfL engagement) and control groups (no CfL)**


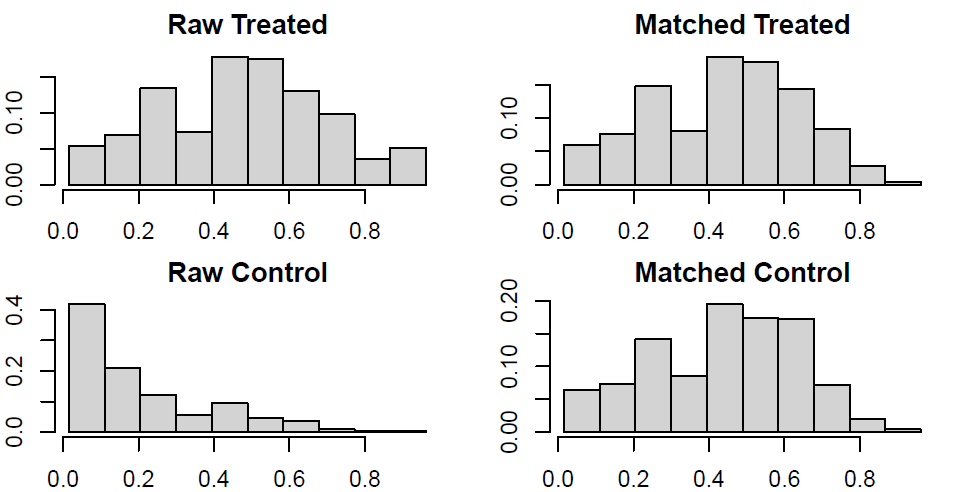


**Fig C. Love Plot, results from balancing the covariates after the matching procedure**

*Note: The red dots indicate the differences between standardized means of covariates in the matched and treated groups for the analytical or the unmatched dataset. The green dots indicate the same for the matched or the adjusted dataset.*


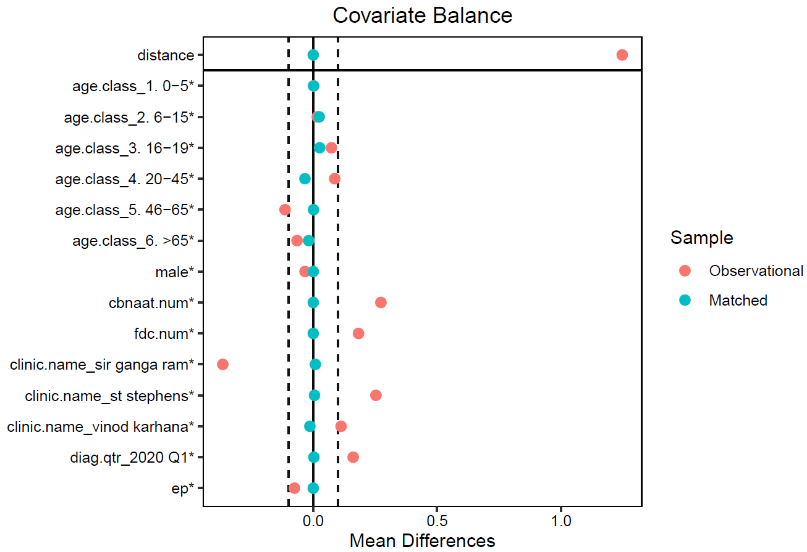


# References

1. Rosenbaum PR, Rubin DB. The central role of the propensity score in observational studies for causal effects. Biometrika. 1983;70:41–55.

2. Winkelmayer WC, Kurth T. Propensity scores: help or hype? Nephrol Dial Transplant. 2004;19:1671–3.

3. Heinrich C, Maffioli A, Vázquez G. A Primer for Applying Propensity-Score Matching. SPD Working Paper. Inter-American Development Bank, Office of Strategic Planning and Development Effectiveness (SPD); 2010.

4. Austin PC, Stuart EA. Moving towards best practice when using inverse probability of treatment weighting (IPTW) using the propensity score to estimate causal treatment effects in observational studies. Stat Med. 2015;34:3661–79.
