# Supplementary material for: Impact of a pilot mHealth intervention on treatment outcomes of TB patients seeking care in the private sector using Propensity Scores Matching—Evidence collated from New Delhi, India
Source: PLOS Digit Health. 2024 Sep 11;3(9):e0000421. doi: 10.1371/journal.pdig.0000421 (PMC11389929; doi:10.1371/journal.pdig.0000421)
Supplement: S3 Appendix — (DOCX) [file pdig.0000421.s003.docx]

# Appendix 3: Regression Results

## Full Model Results; OLS Model; Matched Dataset

**Table A. Results from the full OLS model on matched dataset; N = 745**

| **Dependent Variable: Follow Ups** | |
| --- | --- |
| CfL | 6.417*** (0.573) |
| Xpert Testing | -0.781 (0.781) |
| Free drugs | -0.956 (0.825) |
| Age: 6-15 | -0.317 (2.649) |
| Age: 16-19 | -0.684 (2.665) |
| Age: 20-45 | -0.324 (2.538) |
| Age: 46-65 | -1.744 (2.570) |
| Age: > 65 | -3.206 (2.687) |
| Male | -0.389 (0.484) |
| Extra Pulmonary | 0.885* (0.510) |
| Facility: st stephens | 2.678*** (0.992) |
| Facility: vinod karhana | 4.698*** (0.976) |
| Diag Qtr: 2020 Q1 | 3.990*** (0.701) |
| Facility: st stephens * Diag Qtr 2020 Q1 | -4.599*** (1.208) |
| Facility: vinod karhana * Diag Qtr 2020 Q1 | -6.749*** (1.203) |
| Constant | 8.161*** (2.627) |
| Observations | 745 |
| R | 0.216 |
| Adjusted R | 0.2 |
| Residual Std. Error | 6.359 (df = 729) |
| F Statistic | 13.417*** (df = 15; 729) |
| Note: a) 95% C.I. based on robust standard errors; b) *p<0.1; **p<0.05; ***p<0.01; c) Model was fitted on the matched dataset; d) We have only 745 observations as 199 patients did not have a recorded value for the number of follow ups conducted | |

## Full Model Results; Logistic Model; Matched Dataset

**Table B. Logistic Regression; Impact of CfL on Treatment Outcomes; N = 944**

| **Dependent Variable: Binary Treatment Outcome (Successful=1, Unsuccessful = 0)** | |
| --- | --- |
| CfL | 1.228***(0.34) |
| Xpert Testing | 0.393(0.34) |
| Free drugs | 1.606(1.04) |
| Age: 6-15 | 0.146(1.18) |
| Age: 16-19 | 2.399(1.49) |
| Age: 20-45 | 0.327(1.1) |
| Age: 46-65 | -0.58(1.11) |
| Age: > 65 | -1.326(1.12) |
| Male | -0.525*(0.24) |
| Extra Pulmonary | 0.217(0.23) |
| Facility: st stephens | -1.799***(0.42) |
| Facility: vinod karhana | -1.946***(0.42) |
| Diag Qtr: 2020 Q1 | -0.515(0.35) |
| Facility: st stephens * Diag Qtr 2020 Q1 | 0.472(0.51) |
| Facility: vinod karhana * Diag Qtr 2020 Q1 | 1.241*(0.6) |
| Constant | 3.04**(1.15) |
| Observations | 944 |
| Log Likelihood | -286.184 |
| Akaike Inf. Crit. | 604.37 |
| Note: a) 95% C.I. based on robust standard errors; b) *p<0.1; **p<0.05; ***p<0.01; c) Model was fitted on the matched dataset | |
