# Supplementary material for: Impact of a pilot mHealth intervention on treatment outcomes of TB patients seeking care in the private sector using Propensity Scores Matching—Evidence collated from New Delhi, India
Source: PLOS Digit Health. 2024 Sep 11;3(9):e0000421. doi: 10.1371/journal.pdig.0000421 (PMC11389929; doi:10.1371/journal.pdig.0000421)
Supplement: S4 Appendix — (DOCX) [file pdig.0000421.s004.docx]

# Appendix 4: Sensitivity Analysis

## Results from Simple OLS & Logistic Models; Matched and Unmatched (observational) datasets

**Table A. Sensitivity analysis for OLS regression modelling the impact of CfL engagement on number of follow-ups.**

|  | Coefficient | 95% C.I. | | Number of observations |
| --- | --- | --- | --- | --- |
| Observational | 6.423*** | 5.32 | 7.525 | 774 |
| Matched | 6.417*** | 5.295 | 7.54 | 745 |
| Matched; alternative | 5.757*** | 4.359 | 7.155 | 333 |
| Sir Ganga Ram (only) | 5.083*** | 3.291 | 6.876 | 377 |
| St Stephens (only) | 7.297*** | 5.255 | 9.338 | 203 |
| Vinod Karhana (only) | 6.434*** | 4.48 | 8.389 | 165 |
| Observational; removed LTFU | 6.001*** | 4.89 | 7.111 | 747 |
| Matched; removed LTFU | 6.003*** | 4.874 | 7.132 | 719 |
| Matched; alternative; removed LTFU | 5.329*** | 3.922 | 6.736 | 319 |
| *Note: a) All models control for all potential confounders; b) 95% C.I. based on robust standard errors; c) LTFU refers to patient who were lost to follow-up, d) All facility wise models are fitted on the matched dataset; e) Matched (alternative) refers to a matched dataset which was created by matching using a combination of nearest neighbour (age, free drugs, facility, diagnosing quarter) and exact matching (Xpert testing, gender, extra pulmonary status) methods; f) *p<0.1; **p<0.05; ***p<0.01* | | | | |

**Table B. Sensitivity analysis for logistic regression, modelling the impact of the CfL engagement on treatment outcomes.**

|  | Odds Ratio (OR) | 95% C.I. (OR) | | Number of observations |
| --- | --- | --- | --- | --- |
| Observational | 1.255*** | 1.788 | 6.888 | 989 |
| Matched | 1.228*** | 1.701 | 6.857 | 944 |
| Matched; alternative | 1.181*** | 1.563 | 6.791 | 408 |
| Sir Ganga Ram (only) | 0.425 | 0.349 | 6.702 | 519 |
| St Stephens (only) | 1.250*** | 1.405 | 8.677 | 256 |
| Vinod Karhana (only) | 1.562*** | 1.145 | 19.856 | 169 |
| Observational; removed LTFU | 1.319*** | 1.687 | 8.294 | 962 |
| Matched; removed LTFU | 1.319*** | 1.626 | 8.606 | 918 |
| Matched; alternative; removed LTFU | 1.229*** | 1.391 | 8.405 | 394 |
| *Note: a) All models control for all potential confounders; b) 95% C.I. based on robust standard errors; c) LTFU refers to patient who were lost to follow-up, d) All facility wise models are fitted on the matched dataset; e) Matched (alternative) refers to a matched dataset which was created by matching using a combination of nearest neighbour (age, free drugs, facility, diagnosing quarter) and exact matching (Xpert testing, gender, extra pulmonary status) methods; f) *p<0.1; **p<0.05; ***p<0.01* | | | | |

## Alternative Matching method

Multiple different matching specifications were run to test for the robustness of the model. We report results for one such alternative matching specification. In this alternative specification, we used the nearest neighbor algorithm to match four covariates, 1) age category, 2) free drug status, 3) facility of diagnosis and 4) diagnosing quarter, and exact matching for 1) proportion of males, 2) proportion of extra pulmonary cases, and 3) proportion of patients diagnosed using Xpert testing. The caliper width used was 0.2. This particular matching resulted in 204 pairs (408 observations), relative to 944 observations obtained by way of full matching. The matching resulted in a more similar set of covariates for the two groups, as observed by the p-values obtained for testing difference between two groups.

**Table C. Comparison of descriptive statistics between matched datasets (full matching vs nearest neighbor).**

|  | Matched (Full) | | | Matched (Nearest Neighbor) | | |
| --- | --- | --- | --- | --- | --- | --- |
|  | (N = 944) | | | (N = 408) | | |
| Pilot engagement | no CfL | CfL | *p-value* | no CfL | CfL | *p-value* |
| **Number of patients** | 694 | 250 |  | 694 | 250 |  |
| **Males** | 398 (57%) | 132 (53%) | 0.2 | 109 (53%) | 109 (53%) | >0.9 |
| **Age Category** |  |  |  |  |  | 0.3 |
| 1. 0-5 | 6 (0.9%) | 3 (1.2%) |  | 2 (1.0%) | 3 (1.5%) |  |
| 2. 6-15 | 45 (6.5%) | 20 (8.0%) |  | 12 (5.9%) | 15 (7.4%) |  |
| 3. 16-19 | 51 (7.3%) | 34 (14%) |  | 19 (9.3%) | 32 (16%) |  |
| 4. 20-45 | 334 (48%) | 143 (57%) |  | 121 (59%) | 106 (52%) |  |
| 5. 46-65 | 186 (27%) | 40 (16%) |  | 35 (17%) | 38 (19%) |  |
| 6. >65 | 72 (10%) | 10 (4.0%) |  | 15 (7.4%) | 10 (4.9%) | 0.2 |
| **Age** | 37 (24, 56) | 30 (20, 42) | <0.001 | 29 (22, 45) | 29 (20, 45) | >0.9 |
| **Free drugs** | 41 (5.9%) | 50 (20%) | <0.001 | 39 (19%) | 40 (20%) | >0.9 |
| **Xpert Testing** | 88 (13%) | 97 (39%) | <0.001 | 70 (34%) | 70 (34%) | >0.9 |
| **Extra Pulmonary** | 402 (58%) | 126 (50%) | 0.040 | 101 (50%) | 101 (50%) | <0.001 |
| **Follow Ups** | 11 (4, 16) | 18 (13, 20) | <0.001 | 12 (4, 17) | 18 (13, 21) |  |
| Unknown | 173 | 26 |  | 53 | 22 | 0.9 |
| **Facility** |  |  | <0.001 |  |  |  |
| sir ganga ram | 447 (64%) | 72 (29%) |  | 62 (30%) | 64 (31%) |  |
| st stephens | 143 (21%) | 113 (45%) |  | 84 (41%) | 87 (43%) |  |
| vinod karhana | 104 (15%) | 65 (26%) |  | 58 (28%) | 53 (26%) |  |
| **Successful treatment outcome** | 595 (86%) | 237 (95%) | <0.001 | 170 (83%) | 192 (94%) | <0.001 |
| ***Notes****: 1) The table showcases the numbers segregated by CfL status, and within group percentages for them; 2) For binary/character variables, values represent the number of patients enrolled, and value in parentheses represents share or %; 3) For continuous values, the number represents the median, and the values in parentheses represents the Interquartile Range; 4) Pearson's Chi-squared test and Kruskal-Wallis rank sum test is conducted for p value* | | | | | | |
